# Network approach of the conformational change of c-Src, a tyrosine kinase, by molecular dynamics simulation

**DOI:** 10.1038/s41598-018-23964-5

**Published:** 2018-04-04

**Authors:** Hyun Jung Yoon, Sungmin Lee, Sun Joo Park, Sangwook Wu

**Affiliations:** 1 Department of Physics, Pukyong National University, Busan, 48513 Republic of Korea; 2 Department of Energy Science, Sungkyunkwan University, Suwon, 16419 Republic of Korea; 3 Department of Physics and Institute of Basic Science, Korea University, Seoul, 02841 Republic of Korea; 4 Department of Chemistry, Pukyong National University, Busan, 48513 Republic of Korea

## Abstract

Non-receptor tyrosine kinase c-Src plays a critical role in numerous cellular signalling pathways. Activation of c-Src from its inactive to the active state involves large-scale conformational changes, and is controlled by the phosphorylation state of two major phosphorylation sites, Tyr416 and Tyr527. A detailed mechanism for the entire conformational transition of c-Src via phosphorylation control of Tyr416 and Tyr527 is still elusive. In this study, we investigated the inactive-to-active conformational change of c-Src by targeted molecular dynamics simulation. Based on the simulation, we proposed a dynamical scenario for the activation process of c-Src. A detailed study of the conformational transition pathway based on network analysis suggests that Lys321 plays a key role in the c-Src activation process.

## Introduction

The Src family of non-receptor tyrosine kinases includes c-Src, Fyn, Yes, Blk, Yrk, Fgr, Hck, Lck and Lyn^1^. These tyrosine kinases act as key mediators of various cellular signal transduction processes such as migration, angiogenesis, proliferation, differentiation, survival and immune function^2–4^. The proto-oncogene c-Src, encoded by the Src gene, was first isolated as the normal cellular homologue to the potent avian sarcoma viral transforming oncogene v-Src^5^. Crystal structures of c-Src have shown that its catalytic activity is tightly regulated by autoinhibition via multiple intramolecular interactions^6,7^. All Src family kinases share a similar domain arrangement and consist of a unique N-terminal region, the Src homology 3 (SH3), SH2, linker and tyrosine kinase domains and the regulatory C-terminal tail. c-Src is predominantly inactive under normal cellular circumstances. The SH3 domain binds to the proline-rich region in the linker domain, and the SH2 domain binds to pTyr527, collectively resulting in a closed and inactive protein conformation^8,9^. Many studies have reported the catalytic activation of c-Src in several human cancers^10,11^. These studies show that inactive c-Src changes to an open, active conformation in response to physiological upstream signals.

An important mechanism of c-Src activation involves the control of its phosphorylation status. A major phosphorylation site is Tyr527, which is present in the C-terminal tail of c-Src, but not in v-Src. Dephosphorylation of pTyr527 releases the closed c-Src conformation, resulting in the activation of the SH3, SH2 and tyrosine kinase domains together with an autophosphorylation of Tyr416. This autophosphorylation at Tyr416, which is located in the activation loop of the tyrosine kinase domain, is another essential event in the process. To allow full kinase activity, c-Src must first be dephosphorylated at Tyr527 before it undergoes autophosphorylation at Tyr416^12,13^. Thus, c-Src kinase activity is extremely dependent on the phosphorylation status of the negative-regulatory Tyr527 and the positive-regulatory Tyr416.

Network theory offers a new approach to biological problems^14,15^. For example, Goh *et al*. successfully constructed a network of Mendelian gene–disease associations to identify unknown important genes causing disease^16^. The first step of this approach is to construct a biological network, in which each node is an entity such as a gene and each edge is an interaction or correlation between entities. The constructed network enables a view of the complete set of relationships. In particular, certain measurable properties of these networks facilitate the search for important biological entities. Centrality measures, developed to determine the ranking (i.e., importance) of nodes, have proven effective in uncovering important target genes relevant to disease. In this study, we utilised three centrality measures – the betweenness, closeness and degree centralities – to reveal the important residues controlling the conformational transition of the c-Src protein between the inactive and active conformations, using molecular dynamics simulation.

## Results and Discussions

### Targeted molecular dynamics simulation

Tyr416 is located near the activation loop (Asp404–Glu432) and Tyr527 is located in the tail region of the C-terminus. In the inactive form of c-Src (PDB id: 2SRC)^17^, Tyr527 is phosphorylated (pTyr527) but Tyr416 is not. Conversely, in the active c-Src conformation (PDB id: 1Y57)^18^, Tyr527 is not phosphorylated, whereas Tyr416 is (Supporting Information Fig. S1).

We employed targeted molecular dynamics (TMD) simulation^19^ to study the conformational change of c-Src tyrosine kinase. This method allows the modelling of a continuous transition pathway between two known conformations. TMD simulation can be used to model two directions for the conformational change between the inactive and active states: namely, from inactive to active, or vice versa. In this study, we focused on the activation process, i.e., from the inactive state to the active state.

The phosphorylation at Tyr527 results in multiple intramolecular interactions between pTyr527 and the SH2 domain and between the linker and the SH3 domain. In particular, the pTyr527–SH2 domain interaction plays the role of a lock switch such that the assembly of the domains remains tightly packed together. The main force keeping the assembly of domains packed into a narrow space seems to be electrostatic interactions involving the negative charge of pTyr527. Two positive residues, Arg175 and Lys203, appear to be involved in the electrostatic interaction in the SH2 domain. The positions of Tyr527, Arg175 and Lys203 are shown in Supporting Information Fig. S2.

The side chain of Tyr416 in the activation loop is buried between the two lobes of the kinase domain, effectively protecting Tyr416 from being phosphorylated. The hydrophobic interaction between the inward surface of the α-helix and the N-terminal part of the activation loop also stabilises the inactive conformation.

**Figure 1:**
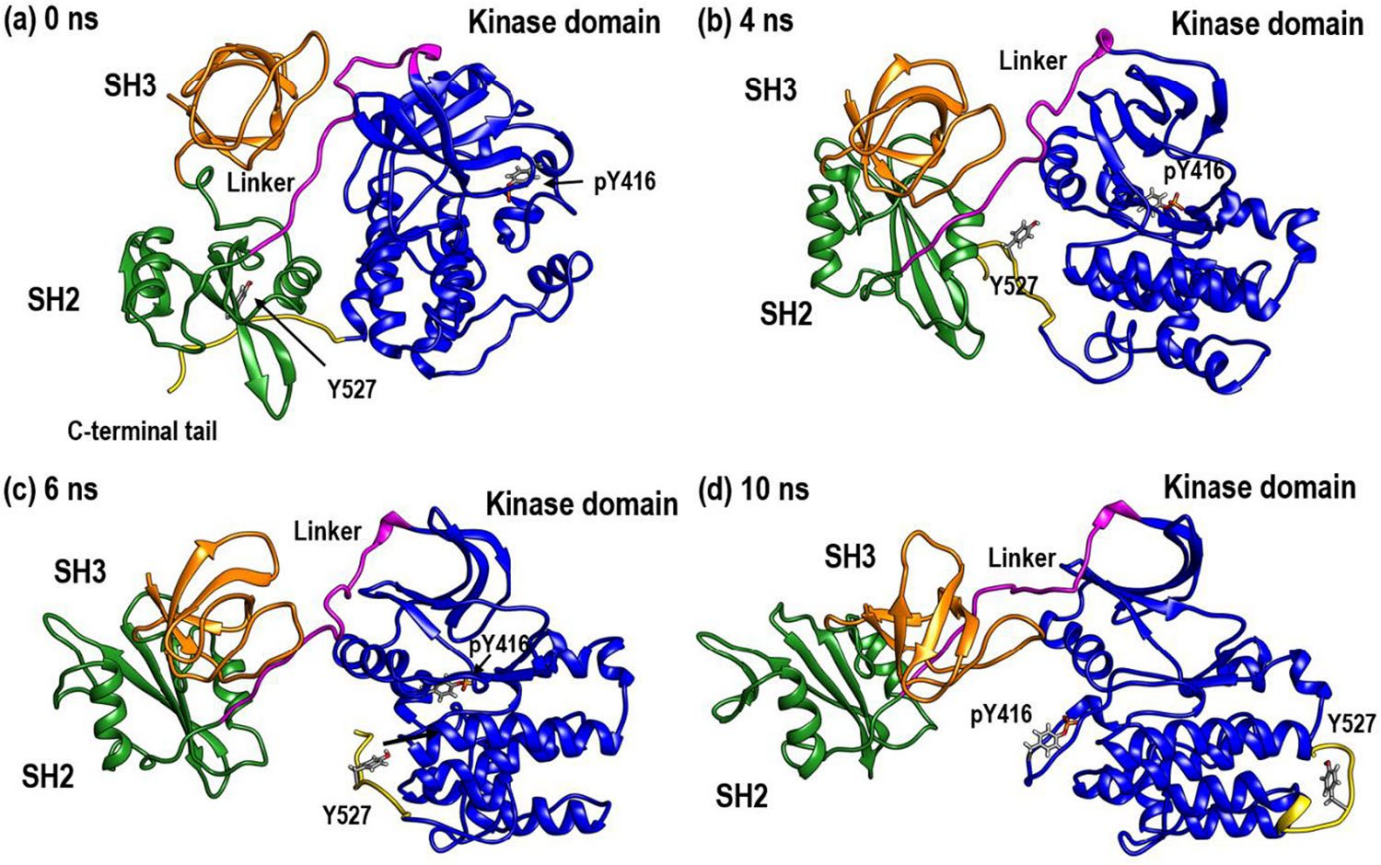
Four snapshots of TMD trajectory of the tyrosine kinase c-Src between 0 and 10 ns. (**a**) 0 ns: Starting structure (inactive conformation) (**b**) 4 ns: Detachment of Tyr527 in the C-terminal tail from the SH2 domain, triggering the transition from inactive to active conformation (**c**) 6 ns: Large-scale conformational change of c-Src (**d**) 10 ns: Target structure (active conformation).

In the transition from the inactive to the active form, the electrostatic interaction between Tyr527 and Arg175/Lys203 becomes weaker. The C-terminal tail, including Tyr527, is completely detached from the SH2 domain during the period of 0–3 ns of the TMD simulation (see Supporting Information Fig. S3). The process of detachment stimulates the unpacking of the domain assembly. The activation processes from the inactive state (0 ns: Fig. 1a) to the active state (10 ns: Fig. 1d) in the TMD simulation are shown in Fig. 1. Between 4 and 6 ns of the simulation, the interaction between Tyr527 (in the C-terminal tail) and its neighbouring positive residues becomes weaker. The detachment of Tyr527 from the SH2 domain triggers the targeted conformational change (Fig. 1b). The detached Tyr527 moves toward the kinase domain (Fig. 1c).

At this stage, the most prominent conformational change occurs at the kinase domain. The secondary structures, including the αC-helix in the kinase domain, rotate significantly. Between 6 and 10 ns, Tyr527 moves away from the SH2 domain and toward the kinase domain. During this time, Tyr416 remains buried beneath the activation loop. By the final stage, Tyr527 has reached the far side of the kinase domain relative to the SH2 domain. The activation loop has also moved from its original position. Tyr416 is now exposed to the surface. The trajectory for conformational transition from the inactive to the active state is shown in Supporting Information Movie 1.

**Figure 2:**
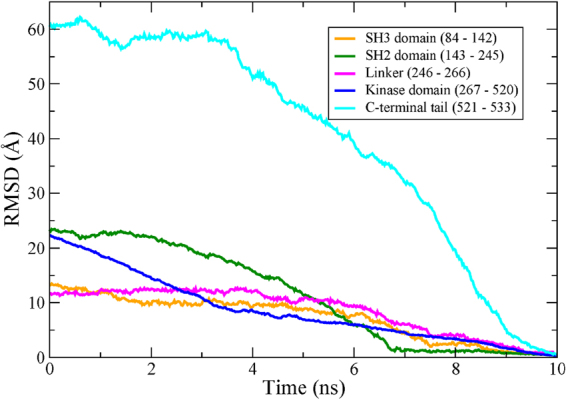
Backbone RMSD values for each domain of the tyrosine kinase c-Src. The starting structure is the inactive conformation and the target structure is the active conformation. The RMSD values of each domain were obtained by aligning the backbone during the TMD trajectory with that of the active conformation. SH3 (orange), SH2 (green), linker (pink), kinase (blue), and C-terminal tail (cyan).

The RMSD of each domain during the 10 ns TMD trajectory is shown in Fig. 2. The domain RMSD values express the deviation of the backbone alignment during the TMD trajectory with respect to the backbone of the active conformation (PDB id: 1Y57). The domain with the largest initial RMSD is the C-terminal tail region (cyan). The RMSD of the C-terminal tail begins at 60 Å and gradually decreases throughout the trajectory.

### Dynamical cross-correlation of c-Src tyrosine kinase

**Figure 3:**
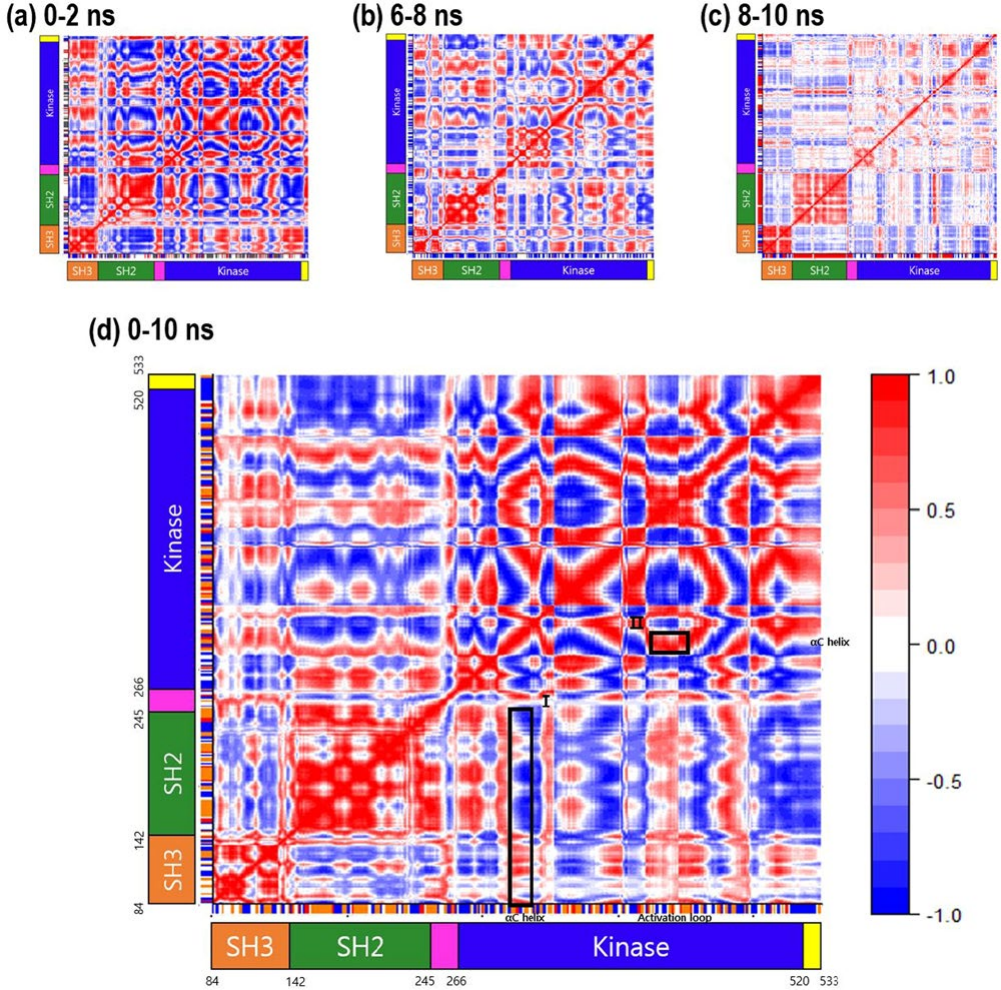
Dynamical cross-correlation maps (DCCMs). Correlation values are in the range of −1 to +1, where positive values mean that two residues are correlated and negative values mean they are anti-correlated.

We calculated a dynamical cross-correlation map (DCCM)^20–22^ to analyse the correlations among the positions of residues during the transition from the inactive to the active conformation. In this map, *C*_*ij*_ ranges between −1 and +1. Positive values mean that two Cα atoms are correlated: they tend to move with same period and phase. Conversely, negative values mean that two Cα atoms are anti-correlated: the two Cα atoms move in the same period but opposite phase. The DCCMs corresponding to four intervals of the TMD simulation are shown in Fig. 3: 0–2 ns (Fig. 3a), 6–8 ns (Fig. 3b), 8–10 ns (Fig. 3c) and 0–10 ns (Fig. 3d). In the DCCMs, the five domains are shown in different colours: SH3 domain (orange), SH2 domain (green), linker (magenta), kinase domain (blue) and C-terminal tail (yellow). During 0–2 ns (Fig. 3a), the intra-domain SH3–SH3 and SH2–SH2 correlations are positive. Conversely, the inter-domain SH3–SH2 correlation is negative. Therefore, during the early stage of the transition, the SH3 and SH2 domains move in an anti-correlated manner. During this stage, the linker region (pink) shows correlated movement with the SH3 domain and anti-correlated movement with the SH2 domain. The C-terminal tail, however, shows the opposite pattern: anti-correlated movement with SH3 and correlated movement with SH2. During 6–8 ns (Fig. 3b), we observe two different correlation patterns for the linker region. The Pro246–Asp258 region of the linker shows correlation with the SH2 domain, whereas the Asp258–Ser266 region shows anti-correlation with the SH2 domain. The activation loop in the kinase domain is anti-correlated with the SH3 domain and correlated with the SH2 domain. Overall, the C-terminal tail shows anti-correlation with the SH2 domain. However, the Lys203–Tyr230 region shows correlation with the SH2 domain. During 8–10 ns, many of the correlation values are close to zero (Fig. 3c). This indicates that the strongest correlation among the residues occurs in the early stage of the transition from the inactive to the active conformation. The DCCM for the entire TMD simulation (0–10 ns) is shown in Fig. 3d. Overall, the residues of the linker region have only a weak correlation with the kinase domain. The αC helix has correlation and anti-correlation with the SH3 and SH2 domains, respectively (denoted as box I in Fig. 3d). In addition, the αC helix has a strong correlation with the activation loop (denoted as box II in Fig. 3d).

### Network analysis

**Figure 4:**
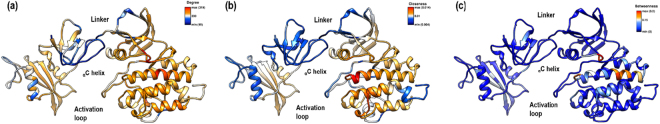
Centrality measures during the 10 ns TMD simulation. (**a**) Degree centrality (**b**) Closeness centrality (**c**) Betweenness centrality. We mapped the centrality measures onto the active conformation.

The degree, closeness and betweenness centralities were measured for the conformational change that occurred during the 10 ns TMD simulation (Fig. 4). The residues with high values of these centralities are not those in the activation loop, linker, or αC-helix. Instead, the residues with high centrality measures are mainly confined to the helix region adjacent to the αC-helix. The residues with the top 5 values of each centrality measure are listed in Table 1.

**Table 1:** Residues with top 5 values of each centrality measure.

	Betweenness	Closeness	Degree
1	Glu320	Val377	Val323
2	Lys321	Arg379	Val402
3	Ala368	Glu378	Lys321
4	Leu322	Met380	Ile370
5	Met380	Thr508	Thr301

**Figure 5:**
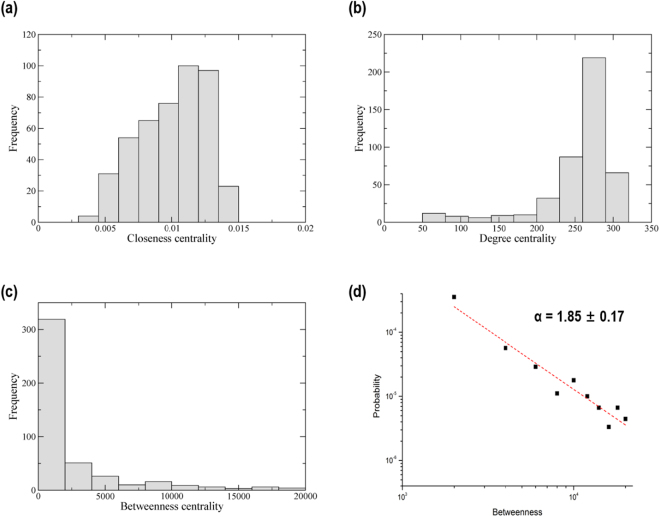
Distributions of the centrality measures during the 10 ns TMD simulation. (**a**) Histogram of closeness centrality (**b**) Histogram of degree centrality (**c**) Histogram of betweenness centrality. (**d**) Power law for the distribution of betweenness centrality.

Histograms of the degree, closeness and betweenness centralities are shown in Fig. 5. In particular, the distribution of the betweenness centrality shows a power-law decrease. By fitting this distribution to the power law^23,24^ *p*(*x*) = *Cx*^−*α*^, the exponent *α* was calculated as 1.85 ± 0.17. The exponent *α* is thus less than 2, indicating that the mean of the betweenness centrality distribution is infinite in the limit of large system size. Values of *α* < 2 are typically found in complex systems, such as the intensity of solar flares (*α* = 1.83)^25^, intensity of wars (*α* = 1.80)^25^ and frequency of family names (*α* = 1.94)^25^. We obtained the same value for the exponent *α* when we calculated the DCCM with a different threshold, *C*_*thr*_ = 0.5 (see Supporting Information Fig. S4).

### Mutation

**Figure 6:**
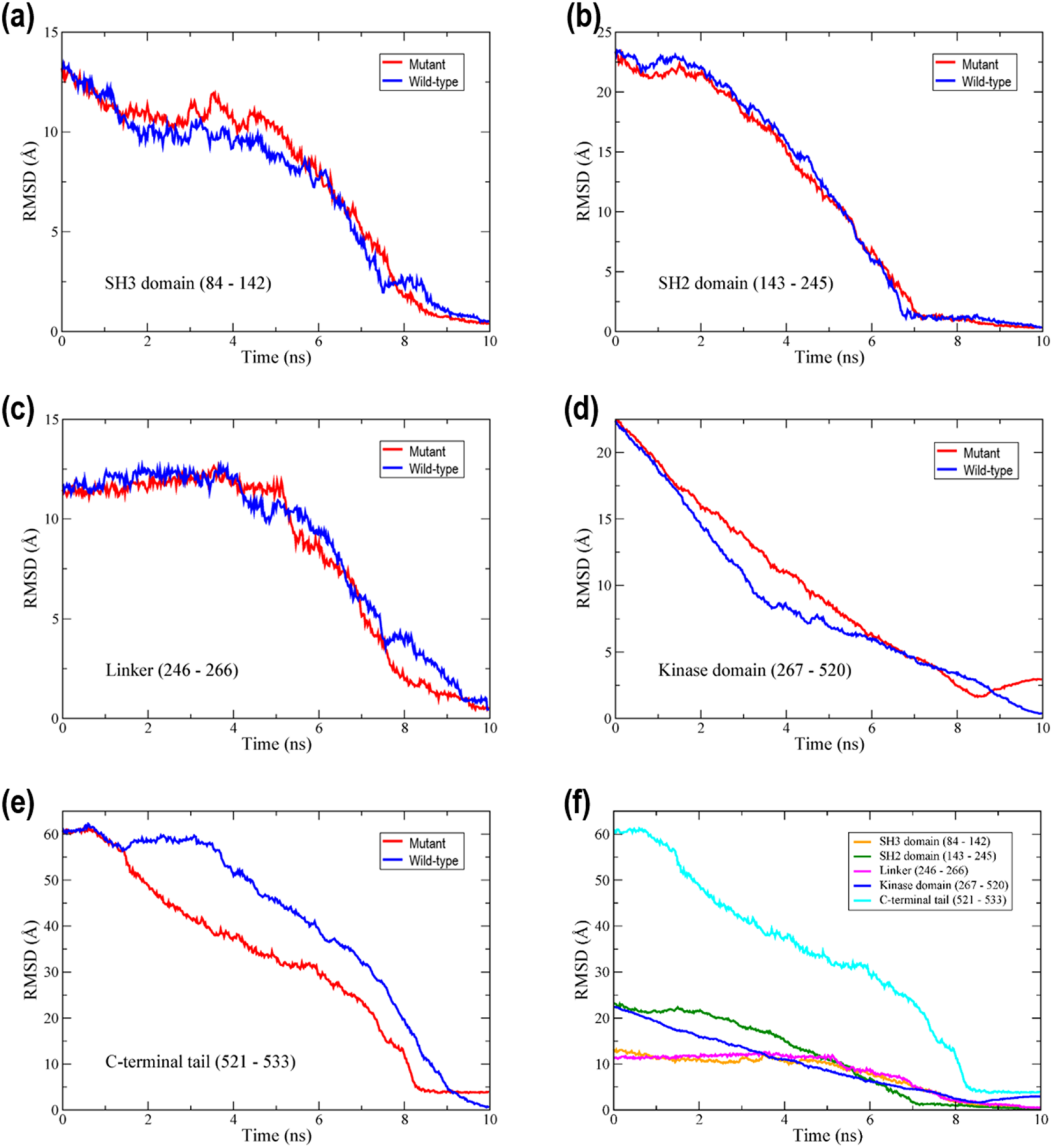
RMSD values for the wild type and the mutant (K321A) during 10 ns TMD simulation for (**a**) SH3 domain, (**b**) SH2 domain, (**c**) Linker, (**d**) Kinase domain, (**e**) C-terminal tail, (f) Overall RMSD values for the mutant.

We conjectured that the residues with high centrality measures would play a critical role in the conformational change of c-Src. To verify this, we selected a residue with high centrality measures, then mutated it, and monitored the conformational transition during a TMD simulation of the mutant c-Src. As the mutation target we selected Lys321, since this residue ranked highly in both the betweenness and degree centralities. Lys321 was mutated into Ala. Subsequently, a 10 ns TMD simulation was performed for the mutant Lys321Ala under the same conditions as previously (i.e., the same spring constant). The RMSD values of the wild type (WT) and mutant for the SH3, SH2, linker, kinase domain (267–520) and C-terminal tail regions are shown, respectively, in Fig. 6a–e. The overall RMSD values for the mutant are shown in Fig. 6f. The conformational change from the inactive to the active conformation failed to reach completion. In particular, the mutation of Lys321 to Ala321 caused notable changes in the linker, kinase domain and C-terminal tail regions during the conformational transition of c-Src. The RMSD values in the linker region are shown in blue for the WT and red for the mutant Lys321Ala (Fig. 6c). For the first 8 ns of the 10 ns TMD simulation, the RMSD values for the WT and mutant show similar trends. However, from 8 ns, the two values diverge. For the mutant, the RMSD value of the linker region sharply decreases at this point. However, for the WT, the RMSD value of the linker region shows a slight recovery at 8 ns before decreasing more gradually. The RMSD values in the kinase domain show a more pronounced difference between the WT and mutant (Fig. 6d). The two RMSD values in the kinase domain diverge at 2 ns, after which the RMSD for the WT decreases more steeply than that for the mutant. The RMSD value of the mutant recovers slightly after 8.5 ns and approaches a final value of approximately 4 Å. This deviation from zero indicates that the kinase domain did not converge to the target structure (the active conformation) under identical TMD simulation conditions as for the WT. An incomplete transition is also observed in the C-terminal tail of the mutant (Fig. 6e). Overall, therefore, the mutation of Lys321 gives rise to an incomplete conformational transition from the inactive to the active conformation.

**Figure 7:**
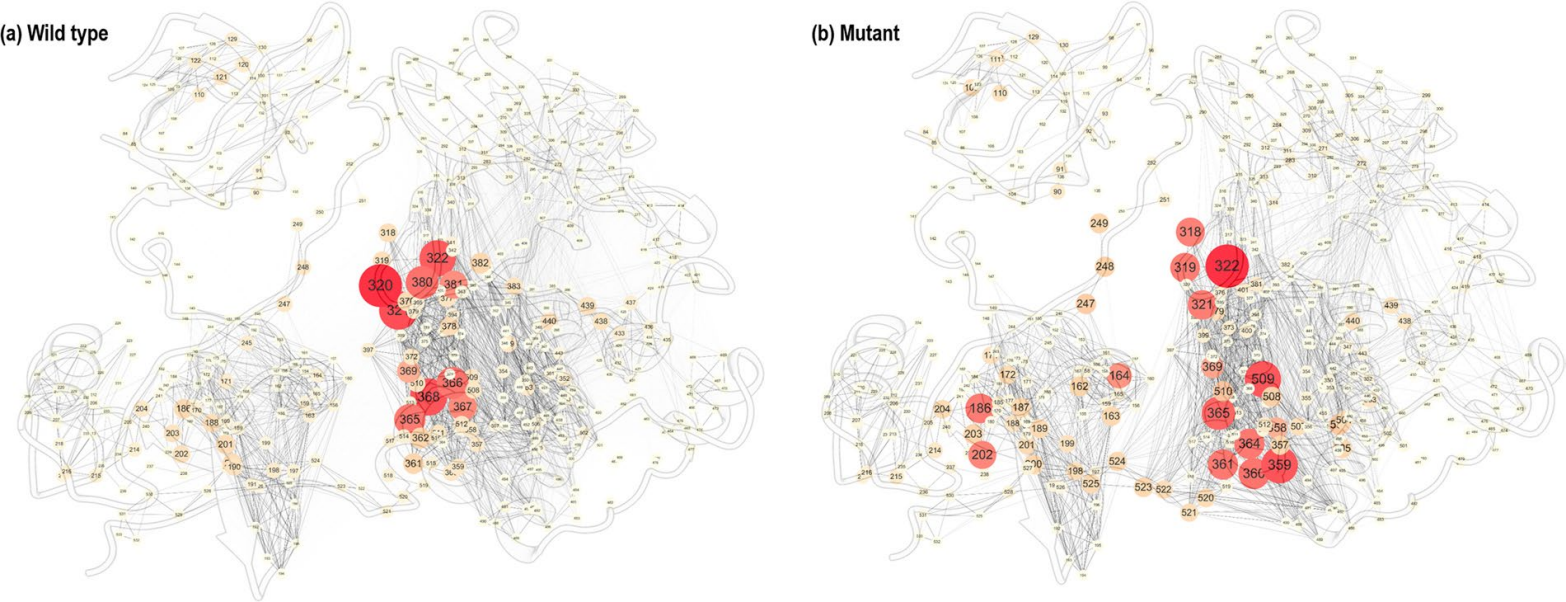
The distribution of the residues with the highest values of the betweenness centrality for (**a**) the WT, (**b**) the mutant during the conformational change. The network topology is the inactive conformation of c-Src (PDB id: 2SRC).

The betweenness centrality values for the WT and the mutant during the conformational change, calculated with *C*_*thr*_ > 0.3, are shown in Fig. 7. The larger the size of each circle, and the more deeply red in colour, the higher the value of the betweenness centrality for the corresponding residue. In Fig. 7, only the edges with weights (*w*_*ij*_) in the range of 0 to 0.2 are shown, for clarity of visualisation. The inactive structure of c-Src (PDB id: 2SRC) was employed as the network topology. Figure 7 shows that the residues with the highest betweenness centralities are heavily concentrated in the kinase domain, with a large number of edges for both the WT and mutant. However, despite sharing the common feature that the residues with the highest betweenness centralities are localised around the kinase domain, there is a distinct difference between the network patterns for the WT and the mutant in detail. For the WT, Arg318–Leu322, Tyr376, Glu378, Met380 and Asn381, which are all located close to Glu320, the residue with the highest value of the betweenness centrality, also show relatively high betweenness centralities. For the mutant, however, the residues with relatively high betweenness centralities are more or less dispersed throughout the kinase domain. The replacement of the charged residue Lys321 by the hydrophobic residue Ala causes the redistribution of the network pattern in the mutant, leading to a different pattern in the conformational change. The residues with the highest values of the betweenness centrality for the WT and the mutant are listed in Supporting Information Table S1. In order to demonstrate the robustness of the betweenness centrality ranking, we analyzed the additional paths generated by WISP (Weighted Implementation of Suboptimal Paths)^26^. The analysis on the additional paths by WISP are shown in Supporting Information (Figs S6, S7, S8, and Table S3).

**Figure 8:**
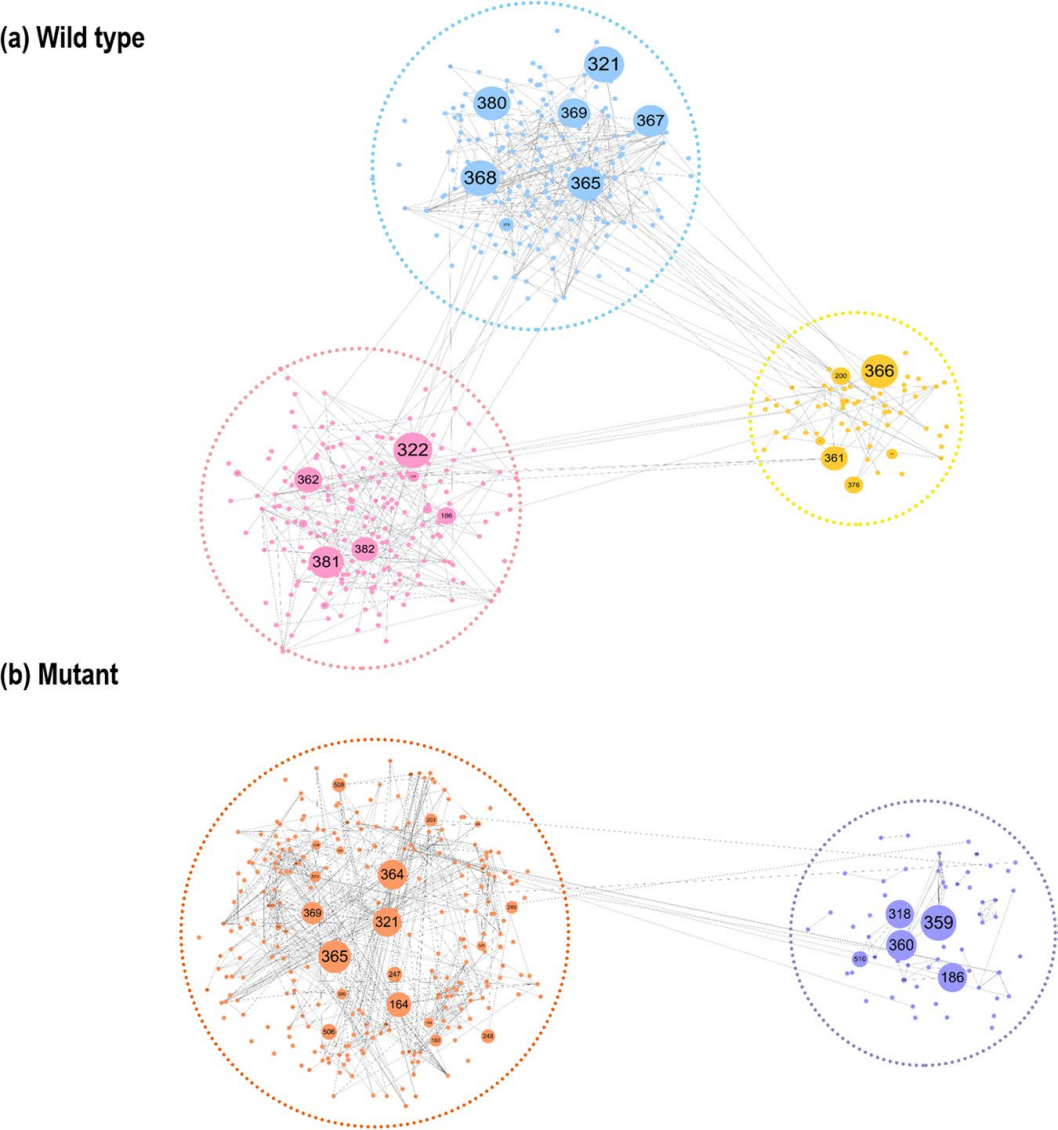
The nodes of the network for (**a**) the WT and (**b**) the mutant, estimated based on the inverse of the weight *w*_*ij*_. The numbers in the nodes denote the corresponding residue of the protein.

The differences in the network patterns of the betweenness centrality between the WT and the mutant are visualised in Fig. 8 using the community structure of the network. Only meaningfully large communities are shown (three communities for the WT and two for the mutant). The community structure was generated with the aid of the topology algorithm in the program Cytoscape^27^. The edge width in the community structure is represented as the inverse of the weight *w*_*ij*_ as defined in Equation 3 in the Methods section. Figure 8 shows the relative “distance” between the nodes (i.e., residues) in the network for the WT and the mutant. By definition, residues within a community have strong correlation while residues in different communities have weak correlation. Importantly, the number of communities is increased from 24 to 130 by the mutation. This indicates that the residues are scattered into smaller communities and the strong correlations between residues are reduced (Table S2). Clearly, the networks for the WT and the mutant differ markedly as a result of this point mutation, implying that Lys321 is a “hub” in the context of the network. The distribution of the residues among each community is listed in Supporting Information Table S2.

## Conclusion

We studied the conformational change of the tyrosine kinase c-Src from the inactive to the active conformation by targeted MD simulation. From the simulation trajectory, a sequential dynamic pathway of the conformational change could be observed. The detachment of Tyr527 in the C-terminal tail from the SH2 domain triggers the conformational change of c-Src. The subsequent domain–domain interactions lead to the exposure of Tyr416, which is initially buried beneath the activation loop in the kinase domain but moves to the surface.

Once it is detached from the SH2 domain, Tyr527 moves from the C-terminal tail toward the kinase domain. Finally, the salt-bridge between Lys295 and Glu310 is formed to complete the conformational change (see Supporting Information Fig. S5).

We quantified the inter/intra-domain correlations of the positions of residues during the conformational change using DCCM. We also constructed a dynamical protein network based on the dynamical interaction among the residues. The degree, closeness and betweenness centralities were measured for the network during the TMD simulation. Notably, we observed that the betweenness distribution follows a power law with the exponent α < 2. By analogy with the “80–20 rule” (wherein 80% of the wealth belongs to the richest 20% of people)^28^, the emergence of an exponent α with a value <2 in the betweenness distribution implies that a very few residues control the conformational transition of c-Src during the conformational change. Indeed, the conformational transition was incomplete for the Lys321Ala mutant. We propose that Lys321 acts as a “hub” in the network during the conformational change. The elimination of this hub leads to a failure of the conformational transition of c-Src. The residues identified as hubs by their high rank in the degree, closeness and betweenness centrality measures would be strong candidates for drug targets to suppress the activation of tyrosine kinases.

## Methods

### Targeted molecular dynamics (TMD) simulation

Performing a TMD simulation requires an external potential, *U*_TMD_, to be defined. *U*_TMD_ is given as^19^

1 $${U}_{TMD}=\frac{k}{2N}{[RMSD(t)-RMS{D}^{\ast }(t)]}^{2}$$

RMSD(*t*) is the root-mean-square deviation (RMSD) of the simulated structure from the target structure at time *t*. RMSD*(*t*) is the RMSD value at time *t* assuming a linear decrease from the initial to the target structure. The inactive form (PDB ID: 2SRC) of c-Src is defined as the initial conformation and the active form (PDB id: 1Y57) as the target conformation. The two conformations were manipulated to have the same number of atoms for the TMD simulation. The spring constant *k* was set as 2500 kcal/mol ·Å^2^ for 3619 atoms (=*N*), excluding hydrogen atoms. We performed the simulations using the NAMD 2.9 package^29^ with the CHARMM 27 force field^30^ and with protein parameters incorporating the CMAP corrections^31^. In TMD, the external force guides the conformational change from the inactive to the active conformation within a reasonable time scale. In our calculation, time was set to 10 ns. We employed as small as possible value of the spring constant (2500 kcal/mol ·Å^2^) to undergo the conformational change within the time scale, minimizing the biasing effect in the system. The TIP3P water model^32^ was employed. The particle mesh Ewald (PME) method was used with a direct space cut-off of 12 Å^33^. The damping coefficient for the Langevin dynamics simulation was 5 ps^−1^. The Nosé–Hoover method was used to maintain constant pressure (1 atm)^34^. The TMD trajectory was performed for 10 ns of simulation time in the NPT ensemble at 310 K. A −2 charge was assigned to the benzene ring of the phosphorylated Tyr416.

### Dynamical cross-correlation analysis

Dynamical cross-correlation^20–22^ is a useful method to analyse the correlation between residues in trajectories of MD simulations.

2 $${C}_{ij}=\frac{ < ({r}_{i}(t)- < {r}_{i}(t) > )({r}_{j}(t)- < {r}_{j}(t) > )}{\sqrt{( < {r}_{i}^{2}(t) > - < {r}_{i}(t){ > }^{2})( < {r}_{j}^{2}(t) > - < {r}_{j}(t){ > }^{2})}}$$

where *r*_*i*_(*t*) and *r*_*j*_(*t*) are the atomic positions of the *i*^th^ and *j*^th^ Cα atoms at time *t*. The quantity *r*_*i*_(t) − <*r*_*i*_(*t*)> corresponds to the fluctuation of the *i*^th^ atom. In a similar way, *r*_*j*_(*t*) − <*r*_*j*_(*t*)> corresponds to the fluctuation of the *j*^th^ atom. We obtained a 450 × 450 correlation map for the conformational changes of all the Cα atoms during the 10 ns TMD simulation.

### Network analysis: centrality measure

Network analysis has become extremely useful in a wide variety of complex systems^35–38^. The quantity *C*_*ij*_ (Eq. 1) in the DCCM can be interpreted as an adjacency matrix. We constructed a network based on it. For this purpose, we defined the weight *w*_*ij*_ of the edge between the nodes *i* and *j* as^39,40^

3 $${w}_{ij}=-log|{C}_{ij}|\,$$

The weight is the probability of information transfer across the edge, as measured by the DCCM obtained from the TMD simulation. Since *C*_*ij*_ has few 0 values the constructed network is almost fully connected. For simplicity of analysis, we introduced the threshold *C*_*thr*_: if *C*_*ij*_ < *C*_*thr*_ the edge between *i* and *j* has no weight (disconnected, *w*_*ij*_ = 0). We performed the following analysis using several values of *C*_*thr*_. Since the results were qualitatively the same in each case, we present the results only for *C*_*thr*_ = 0.3. In the constructed network, each node corresponds to a Cα atom and each edge is an information transfer probability (i.e., cross-correlation).

To identify and quantify the nodes that occupy critical positions in a network, several centrality measures have been proposed, including the degree, betweenness, eigenvector and closeness centralities^40–42^. In this work, we used three centrality measures based on the DCCM: degree centrality, closeness centrality and betweenness centrality. The degree centrality measures the number of edges incident on a node in a network, thus expressing the “popularity” of the node^41,42^.

4 $${C}_{D}({v}_{i})={d}_{i}=\sum _{j}{A}_{ij}$$

where *A*_*ij*_ is the adjacency matrix: if *w*_*ij*_ > 0 then *A*_*ij*_ = 1, otherwise *A*_*ij*_ = 0. The closeness centrality is defined as the average length of the shortest paths between a node and all the other nodes in a network. This is a measure of how long it will take information to spread from a given node to the other nodes. The closeness centrality is defined as^41,42^

5 $${C}_{C}({v}_{i})=\frac{n-1}{{\sum }_{j\ne i}g({v}_{i},\,{v}_{j})}$$

where *g*(*v*_*i*_, *v*_*j*_) is the shortest path with a weight between two nodes *i* and *j*. The betweenness centrality is a measure of how many information pathways flow through a node in a network. The betweenness of a node *i* is the fraction of the shortest paths between pairs of nodes that pass through node *i*. It is defined as^41–43^

6 $${b}_{i}=\frac{{\sum }_{s < t}{g}_{i}^{st}/{n}_{st}}{\frac{1}{2}n(n-1)}\,$$

where ${g}_{i}^{st}$ is the number of shortest path*s* from *s* to *t* with a weight that pass through node *i* and *n*_*st*_ is the total number of shortest paths from *t*o *s* to *t*. The three centrality measures were obtained using Bio3d^44–46^.

The images of the nodes of the network (Fig. 8) were generated with the “refuse force directed layout” algorithm in Cytoscape^27^.

## Electronic supplementary material

Supporting Information

TMD trajectory video of the tyrosine kinase c-Src between 0 and 10 ns
