# Supplementary material for: Network approach of the conformational change of c-Src, a tyrosine kinase, by molecular dynamics simulation
Source: Sci Rep. 2018 Apr 4;8:5673. doi: 10.1038/s41598-018-23964-5 (PMC5884825; doi:10.1038/s41598-018-23964-5)
Supplement: Supplementary file 1 — Supporting Information [file 41598_2018_23964_MOESM1_ESM.docx]

**Supporting Information**

**Network approach of the conformational change of c-Src, a tyrosine kinase, by molecular dynamics simulation**

Hyun Jung Yoon^1^, Sungmin Lee^2,3^, Sun Joo Park^4,*^, Sangwook Wu^1,*^

*^1^Department of Physics, Pukyong National University,Busan, 48513, Republic of Korea*

*^2^Department of Energy Science, Sungkyunkwan University, Suwon, 16419, Republic of Korea*

*^3^Department of Physics and Institute of Basic Science, Korea University,*

*Seoul, 02841, Republic of Korea*

*^4^Department of Chemistry, Pukyong National University,Busan, 48513, Republic of Korea*

**
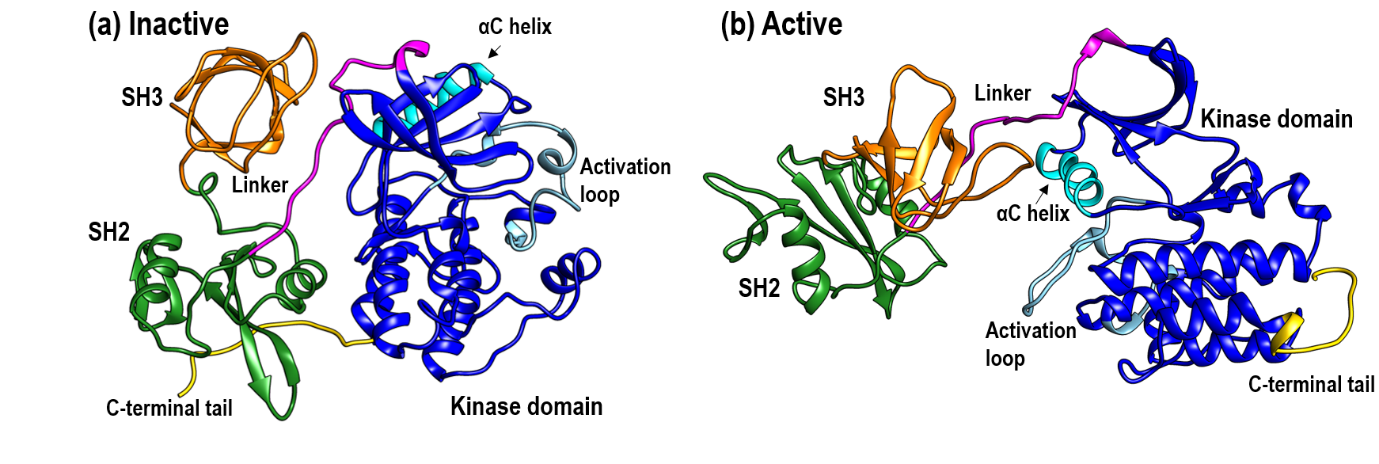
**

**Figure S1.** Two X-ray crystal structures of the tyrosine kinase c-Src: Inactive conformation (PDB id: 2SRC) and active conformation (PDB id: 1Y57).


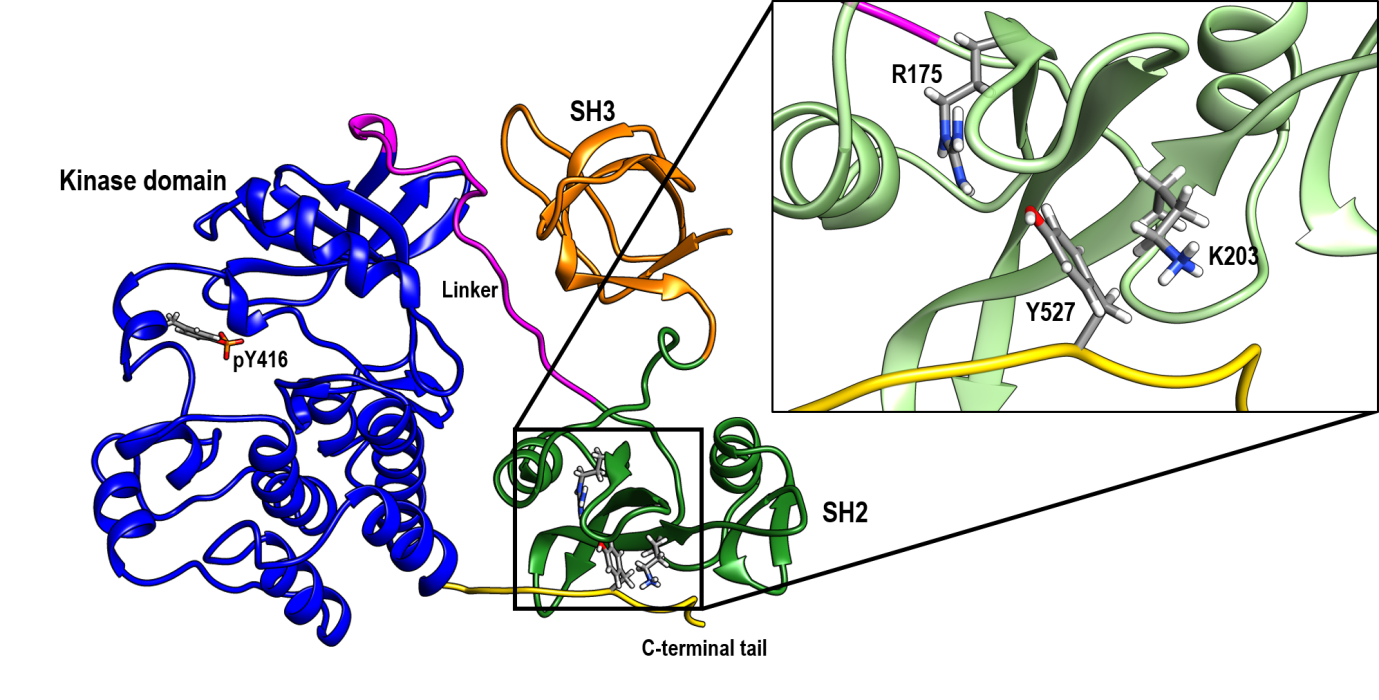


**Figure. S2** The relative positions of the Tyr527, Arg175, and Lys203 in the SH2 domain of the inactive conformation.


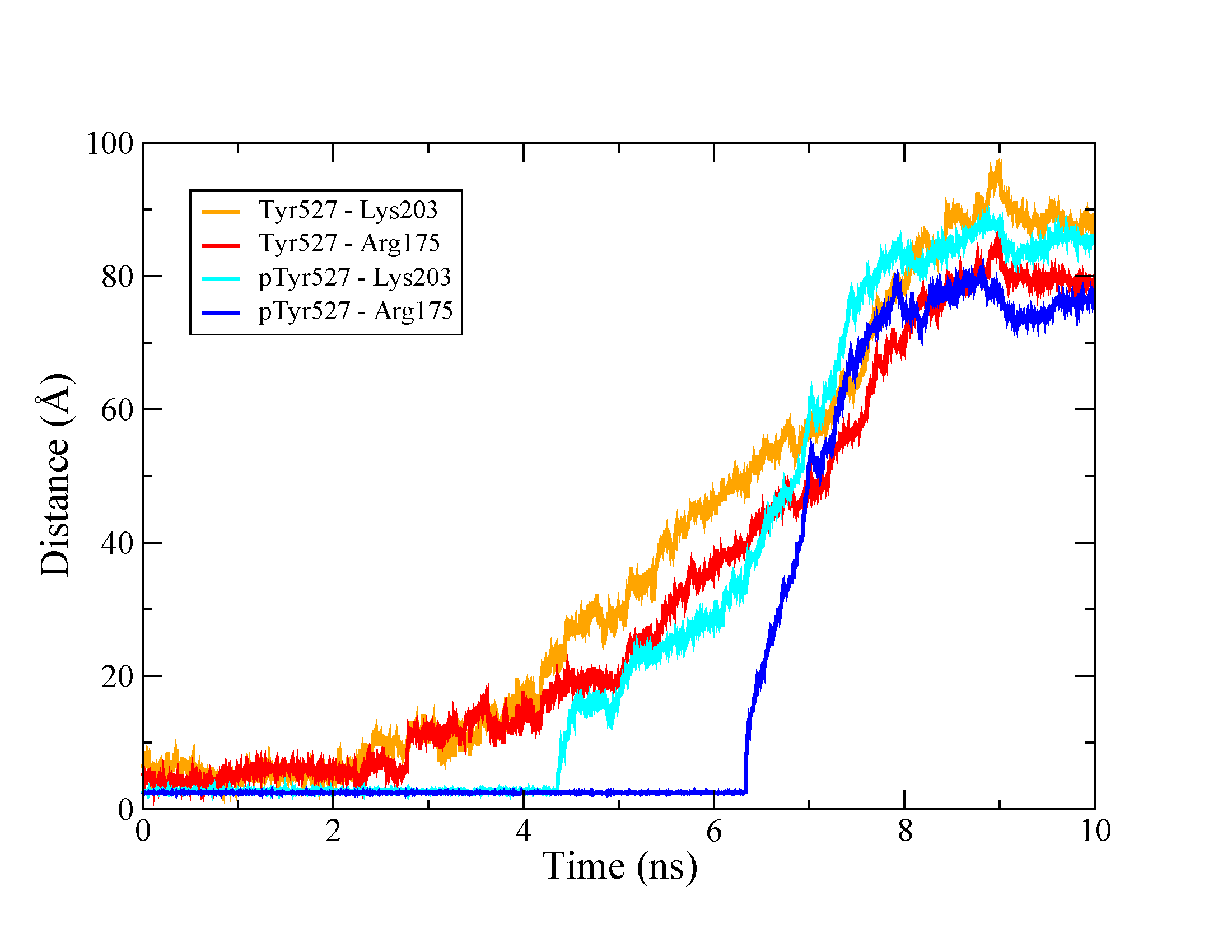


**Figure. S3** The distance between Tyr527 and Lys203 (orange) and Tyr527 and Arg175 (red). The interactions between the Tyr527 and Arg175/Lys203 are more prolonged when Tyr527 is phosphorylated.


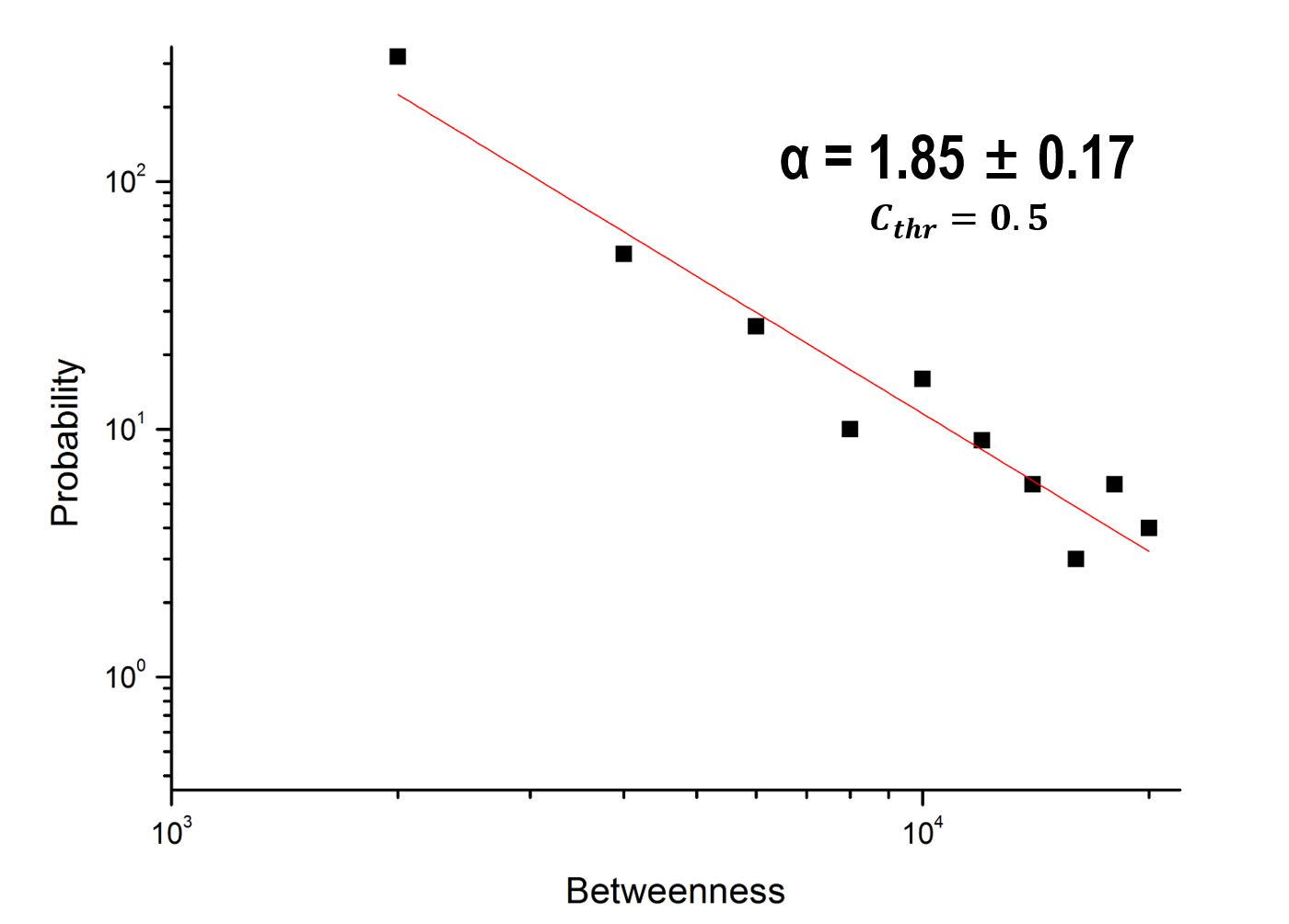


**Figure. S4** The critical exponent of the betweenness centrality obtained from C_thr_=0.5 is the same with the value with C_thr_=0.3.


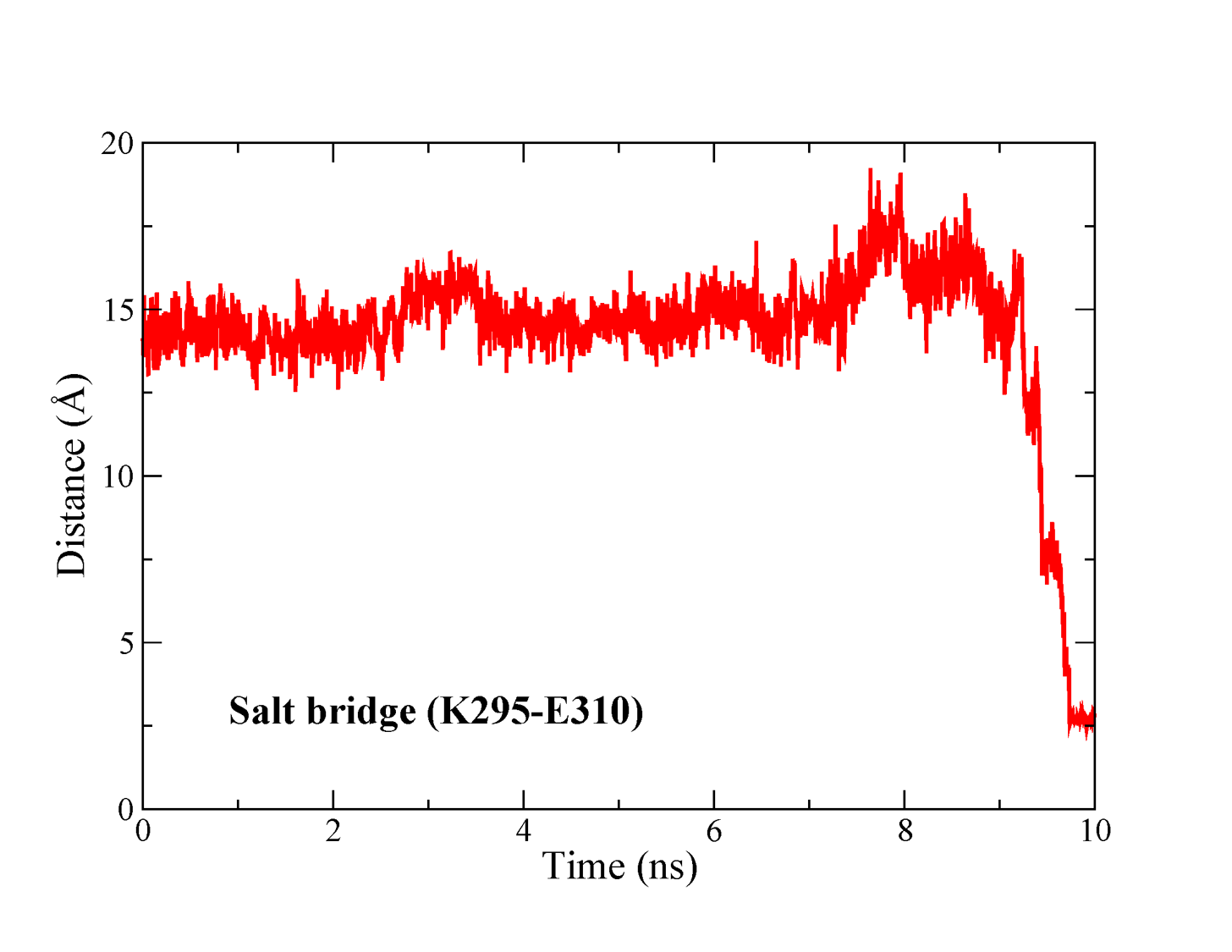


**Figure. S5** The salt bridge between Lys295 and Glu310 during 10 ns TMD simulation from the inactive to the active conformation.

| WT | | Mutant | |
| --- | --- | --- | --- |
| Residue | Betweenness  (Unnormalized) | Residue | Betweenness  (Unnormalized) |
| GLU 320 | 18994 | LEU 322 | 16303 |
| LYS 321 | 18478 | ARG 359 | 14878 |
| ALA 368 | 18329 | PHE 509 | 14819 |
| LEU 322 | 18229 | ASP 365 | 14205 |
| MET 380 | 17841 | LEU 360 | 13719 |
| MET 366 | 17734 | PRO 361 | 13543 |
| ASP 365 | 17660 | VAL 364 | 13539 |
| ALA 367 | 17307 | LEU 186 | 13418 |
| ASN 381 | 17286 | HSD 319 | 13412 |
| GLN 369 | 16720 | ALA 321 | 13362 |
| GLN 362 | 15575 | ARG 318 | 13250 |
| PRO 361 | 15276 | TYR 202 | 13065 |
| TYR 382 | 15267 | ASN 164 | 12473 |
| LYS 200 | 13420 | GLN 369 | 11907 |
| LEU 186 | 13386 | LEU 358 | 11369 |
| TYR 376 | 13333 | GLU 510 | 10550 |
| HSD 201 | 13149 | ARG 506 | 10473 |
| GLU 378 | 12334 | THR 247 | 10364 |
| ARG 318 | 12303 | SER 248 | 10089 |
| HSD 319 | 11676 | THR 508 | 10082 |
| VAL 188 | 11555 | LYS 203 | 9865 |
| PHE 439 | 11101 | LYS 249 | 9785 |
| TYR 202 | 10955 | LEU 162 | 9695 |
| THR 440 | 10830 | THR 523 | 9495 |
| LYS 203 | 10689 | SER 187 | 9439 |
| VAL 383 | 10544 | GLU 505 | 9345 |
| ARG 438 | 10225 | LYS 200 | 9331 |
| GLU 510 | 10096 | LEU 163 | 9223 |
| ASP 190 | 9975 | TYR 357 | 9120 |
| PHE 509 | 9884 | GLU 524 | 9111 |

**Table S1.** Residues with the higher values in the betweenness centrality for the WT and the mutant.

**(a) WT**

Network nodes 450 / edges 68366Number of Communities 24 / Inter-community edges 181

| Wild Type | Residue number |
| --- | --- |
| Community 1 | 84, 85, 87, 88, 89, 97, 107, 108, 109, 110, 111, 112, 116, 120, 121, 122, 129, 130, 131, 132, 134, 136, 137, 138, 142, 146, 149, 151, 152, 174, 175, 176, 177, 178, 179, 184, 185, 186, 202, 203, 209, 210, 221, 222, 224, 225, 230, 231, 232, 233, 234, 235, 236, 237, 251, 252, 258, 259, 260, 264, 265, 267, 268, 269, 270, 271, 272, 273, 274, 277, 278, 279, 281, 282, 286, 287, 289, 290, 291, 292, 294, 296, 297, 298, 303, 304, 311, 312, 313, 319, **322**, 324, 325, 326, 329, 330, 331, 340, 341, 342, 358, 359, 360, 362, 363, 371, 372, 373, **381**, 382, 383, 384, 388, 392, 393, 394, 395, 396, 397, 398, 404, 405, 408, 411, 412, 413, 414, 416, 417, 418, 419, 428, 440, 441, 442, 443, 444, 448, 449, 453, 454, 455, 456, 461, 462, 466, 467, 472, 473, 474, 475, 476, 477, 479, 482, 483, 484, 485, 486, 487, 493, 494, 495, 496, 501, 502, 503, 504, 511, 512, 513, 514, 515, 516, 517, 518, 519, 520, 521, 522, 523, 524, 525, 526, 527, 528, 529, 530 |
| Community 2 | 86, 115, 119, 133, 140, 143, 148, 150, 153, 154, 155, 156, 173, 187, 188, 200, 212, 223, 238, 239, 240, 241, 261, 263, 276, 283, 284, 302, 309, 310, 337, 338, 339, 347, 348, 351, 352, 361, 364, **366**, 374, 375, 376, 385, 390, 399, 400, 406, 420, 424, 426, 430, 438, 439, 451, 460, 488, 497, 498, 507, 508, 509 |
| Community 4 | 91, 92, 98, 99, 100, 104, 105, 106, 117, 118, 123, 124, 125, 126, 127, 128, 135, 139, 141, 144, 145, 157, 158, 159, 160, 161, 162, 163, 164, 165, 166, 167, 168, 169, 170, 171, 172, 180, 181, 182, 183, 189, 190, 191, 192, 193, 194, 195, 196, 197, 198, 199, 204, 205, 206, 207, 208, 213, 214, 215, 216, 217, 218, 219, 220, 226, 227, 228, 229, 242, 243, 244, 245, 246, 247, 248, 249, 250, 256, 257, 262, 266, 275, 280, 285, 288, 293, 295, 299, 300, 301, 305, 306, 307, 308, 314, 315, 316, 317, **321**, 323, 327, 328, 332, 333, 334, 335, 343, 344, 345, 346, 349, 350, 353, 354, 355, 356, 357, **365**, **367**, **368**, **369**, 370, 377, 378, 379, **380**, 386, 387, 389, 391, 401, 402, 403, 407, 409, 410, 421, 422, 423, 425, 427, 429, 431, 432, 433, 434, 435, 436, 437, 445, 446, 447, 450, 452, 457, 458, 459, 463, 464, 465, 468, 469, 470, 471, 478, 480, 481, 489, 490, 491, 492, 499, 500, 505, 506, 531, 532, 533 |

Community 5 : 93 Numbers denote residues of c-Src tyrosine kinase.

**(b) Mutant (K321A)**

Network nodes 450 / edges 68211

Number of Communities 130 / Inter-community edges 5684

| Mutation | **Residue number** |
| --- | --- |
| Community 2 | 85, 109, 110, 111, 112, 120, 121, 149, 151, 152, 174, 175, **186**, 201, 231, 234, 237, 261, 262, 267, 268, 270, 271, 273, 277, 279, 294, 296, 302, 310, 318, 325, 331, 337, **359**, **360**, 373, 392, 395, 396, 397, 398, 406, 415, 416, 418, 440, 443, 448, 449, 453, 466, 472, 473, 487, 496, 501, 502, 503, 510, 512 |
| Community 4 | 87, 88, 90, 91, 98, 104, 105, 107, 108, 116, 118, 122, 123, 124, 125, 126, 127, 128, 129, 130, 131, 132, 134, 135, 136, 137, 139, 140, 141, 142, 144, 145, 160, 161, 162, 163, 164, 165, 166, 167, 168, 169, 170, 171, 177, 178, 179, 180, 181, 182, 183, 184, 191, 192, 193, 194, 195, 196, 197, 203, 204, 205, 206, 207, 208, 209, 210, 211, 212, 213, 214, 215, 216, 217, 218, 219, 220, 221, 222, 223, 224, 225, 226, 227, 228, 229, 230, 232, 233, 235, 242, 243, 244, 245, 246, 247, 248, 249, 250, 251, 252, 253, 254, 255, 257, 258, 260, 263, 264, 266, 272, 274, 275, 278, 281, 285, 286, 288, 291, 292, 293, 295, 297, 298, 299, 300, 301, 303, 304, 305, 306, 307, 313, 314, 315, 316, 320, **321**, 323, 324, 329, 330, 333, 334, 335, 336, 342, 343, 344, 345, 346, 347, 349, 350, 353, 354, 355, 356, 357, **364**, **365**, 366, 367, 368, 369, 370, 377, 378, 379, 380, 381, 386, 387, 388, 389, 391, 401, 402, 403, 404, 405, 408, 409, 410, 411, 412, 421, 422, 423, 425, 426, 427, 428, 429, 430, 431, 432, 433, 434, 435, 436, 437, 438, 439, 442, 445, 446, 447, 450, 454, 457, 458, 459, 462, 463, 464, 465, 468, 469, 470, 471, 475, 476, 477, 478, 479, 480, 481, 482, 484, 485, 489, 490, 491, 492, 493, 498, 499, 500, 505, 506, 507, 508, 516, 517, 518, 519, 520, 521, 522, 523, 524, 525, 526, 527, 528, 529, 530, 531, 532, 533 |

Community 36 : 159, Community 44 : 190, Community 45 : 198

**Table S2.** Residues belonging to community network (a) WT and (b) mutant

**Analysis on the additional paths generated by WISP**

We fixed two targeted atoms for the analysis of the shortest pathways generated by WISP. We chose the two atoms, W260 and Y527, which are known as the important residues controlling the conformational change. W260 is positioned in the linker region and Y527 is the one of the major phosphorylation sites. The shortest pathway between W260 and Y527 during 8-9 ns time interval (Fig. S6(A)) and 9-10 ns (Fig. S6(B)) searched by WISP. In the Fig.S6, Lys321 is located in the middle of the shortest pathway between W260 and Y527.


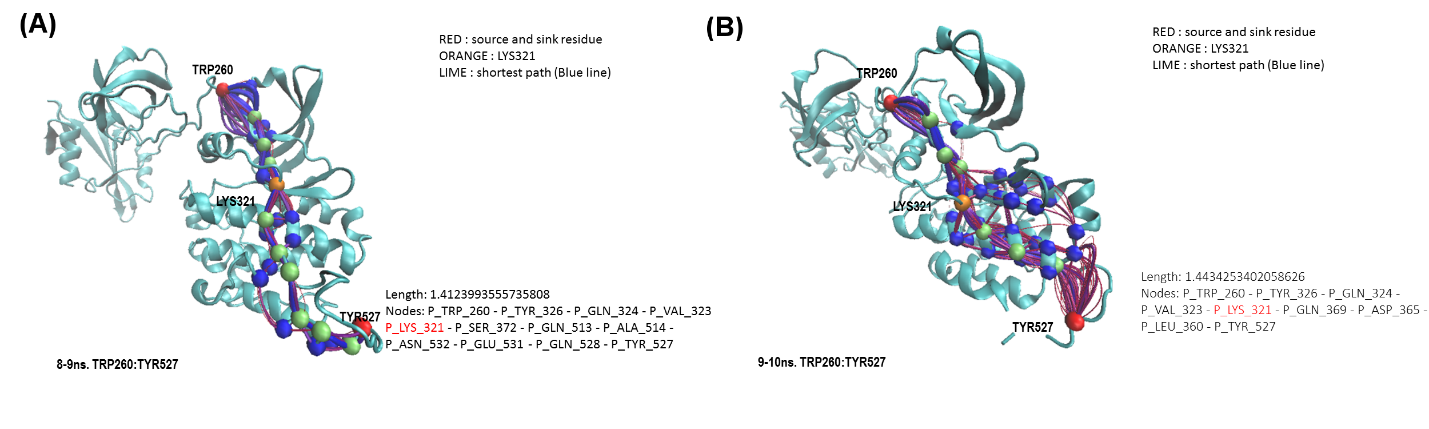


**Fig.S6** Shortest pathway between TRP260 and TYR527 during (A) 8-9 ns time interval (B) 9-10 ns in the conformational change from the inactive to active conformation. Red atoms correspond to W260 and Y527. Lys321 is shown (Orange). The residues in the shortest path are shown (Lyme). Among the paths, the shortest paths are shown as thick blue line.

**New shortest pathways by deleting three residues Glu320-Lys321-Leu322 between W260 and Y527.**


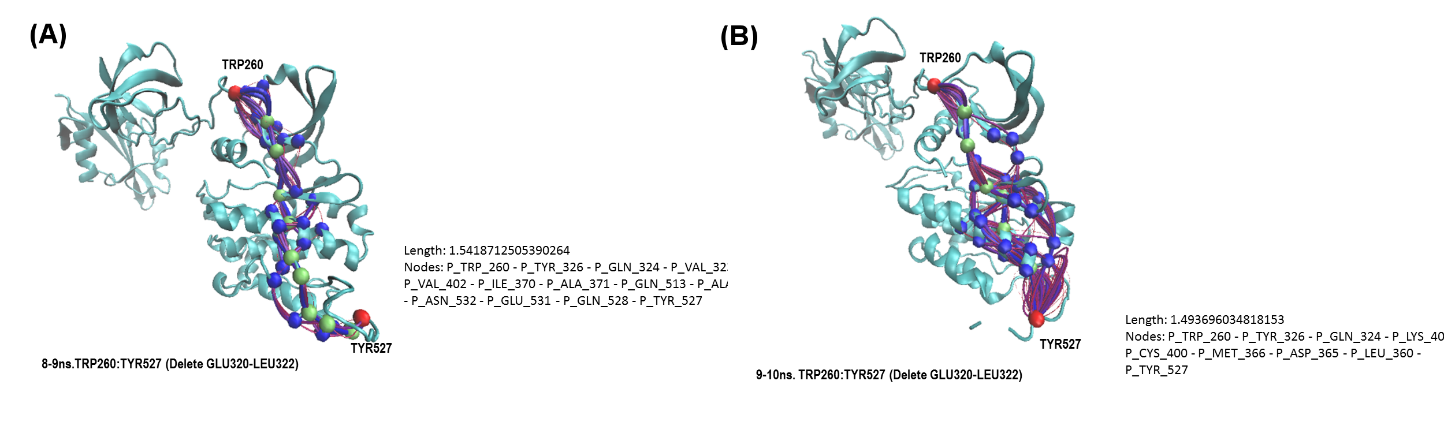


**Fig.S7** Shortest pathway between TRP260 and TYR527 during (A) 8-9 ns time interval (B) 9-10 ns in the conformational change from the inactive to active conformation with the deletion of three residues (Glu320-Lys321-Leu322). Red atoms correspond to W260 and Y527. The residues in the shortest path are shown (Lyme). Among the paths, the shortest paths are shown as thick blue line.

A comparison of shortest pathways between the WT and deleted residues during 8-9 ns shows that the deletion of Lys321, as a “hub” in our network, changed the shortest pathways by increasing the intermediates by three more residues. On the other hand, the comparison of the shortest pathways for both WT and deleted residues shows that the intermediates are changed, but the number of intermediates are not changed. The shortest pathways are summarized in the Table S3.

|  | Shortest Pathway Between W260 and Y527 |
| --- | --- |
| 8-9 ns (WT) | W260-Y326-Q324-V323-**K321**-Q513-A514-N532-E531-Q528-Y527 |
| 8-9 ns (deletion of E320/K321/L322) | W260-Y326-Q324-V323-**V402-I370-A371**-Q513-A514-N532-E531-Q528-Y527 |
| 9-10 ns (WT) | W260-Y326-Q324-**V323-K321-Q369**-D365-L360-Y527 |
| 9-10 ns (deletion of E320/K321/L322) | W260-Y326-Q324-**K401-C400-M366**-D365- L360-Y527 |

**Table S3**. Shortest pathways searched by WISP for during 8-9 ns/8-9ns (deletion of Glu320-Lys321-Leu322)/9-10ns/9-10ns (deletion of Glu320-Lys321-Leu322).

**Histogram of the shortest pathway distribution for 500 paths between W260 and Y527**

Histogram analysis shows that the deletion of Lys321 during 8-9 ns increased the path length (maximum value) from ~ 1.5 to ~ 1.65. On the other hand, the path length difference due to a deletion of Lys321 during 9-10 ns was small. The path length during 8-9 ns time interval is more sensitive to a deletion of Lys321 than the path length during 9-10 ns time interval. This demonstrates that Lys321 plays a critical role in the conformational change from the inactive to the active form during 8-9 ns, which is consistent with the mutation Lys321Ala simulation result in the main text.


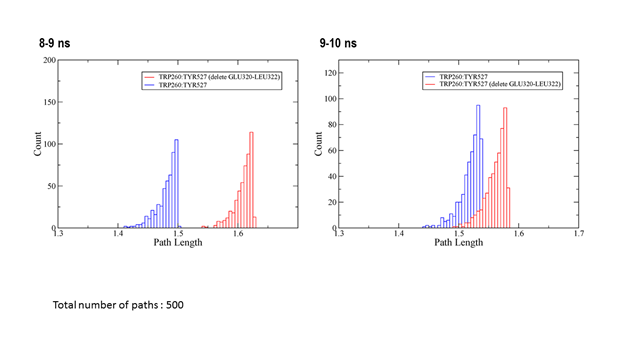


**Fig.S8** Histogram of the distribution of the (A) shortest pathways during 8-9 ns (blue) /8-9ns (deletion of Glu320-Lys321-Leu322) (red) (B) 9-10ns (blue)/9-10ns (deletion of Glu320-Lys321-Leu322) (red).
